# Disseminated Histoplasmosis in an Immunocompetent Patient After COVID-19 Pneumonia

**DOI:** 10.7759/cureus.17269

**Published:** 2021-08-18

**Authors:** Matthew Taylor, Arjun Ghodasara, Ali Ismail, Umair Gauhar, Karim El-Kersh

**Affiliations:** 1 Pulmonary and Critical Care, University of Louisville, Louisville, USA; 2 Pathology and Laboratory Medicine, University of Louisville, Louisville, USA; 3 Interventional Pulmonary, University of Louisville, Louisville, USA; 4 Pulmonary and Critical Care, University of Nebraska Medical Center, Omaha, USA

**Keywords:** covid-19, disseminated histoplasmosis, fungal, immunocompetent, dexamethasone

## Abstract

Disseminated histoplasmosis can occur in immunocompromised patients such as in HIV disease and patients with medication-induced immunosuppression. Most of these patients present with fever, weight loss, hepatosplenomegaly, lymphadenopathy, and pancytopenia. There are increasing reports of coronavirus disease 2019 (COVID-19) pneumonia associated with fungal infections including aspergillus and mucormycosis. It is not typical for immunocompetent patients to present with disseminated fungal disease. We herein report a case of a 50-year-old immunocompetent male with a recent recovery from COVID-19 pneumonia who presented with fever and pancytopenia. Chest computed tomography (CT) demonstrated new-onset right upper lobe lung mass, subcarinal lymphadenopathy, and splenomegaly. Mediastinal lymph nodes and bone marrow biopsies were performed, and the patient was diagnosed with disseminated histoplasmosis. The association between COVID-19 pneumonia and fungal infections is increasingly reported. Diagnosis requires a high index of suspicion, especially in immunocompetent patients.

## Introduction

Coronavirus disease 2019 (COVID-19) pneumonia-associated fungal infections are well documented with risk factors including ICU prolonged length of stay, neutropenia, hematological malignancies treated with chemotherapy, transplantation, and other immunocompromised states like HIV [1,2]. Disseminated histoplasmosis is often seen in immunosuppressed patients, and can present with multisystem involvement including the lungs, skin, gastrointestinal tract, and bone marrow [3-5]. Disseminated histoplasmosis in patients with post-COVID-19 pneumonia is rare and is difficult to diagnose [6]. A disseminated disease requires treatment and with amphotericin B for one to two weeks followed by azole antifungal therapy [7].

## Case presentation

**Figure 1:**
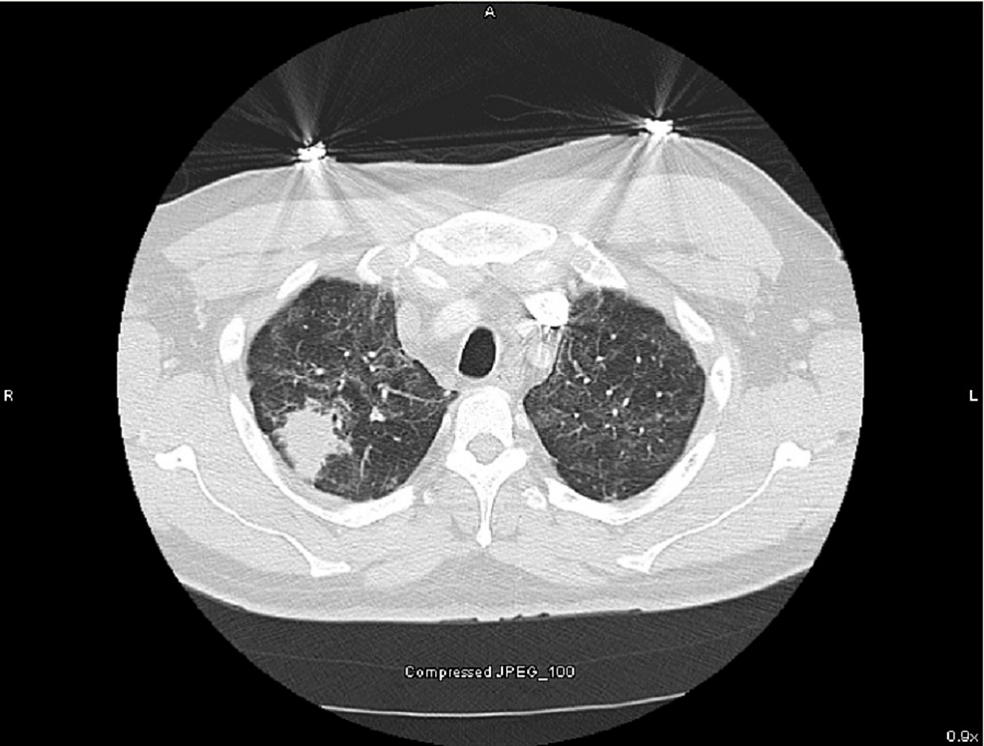
CT of the chest with right upper lobe (RUL) mass.

**Figure 2:**
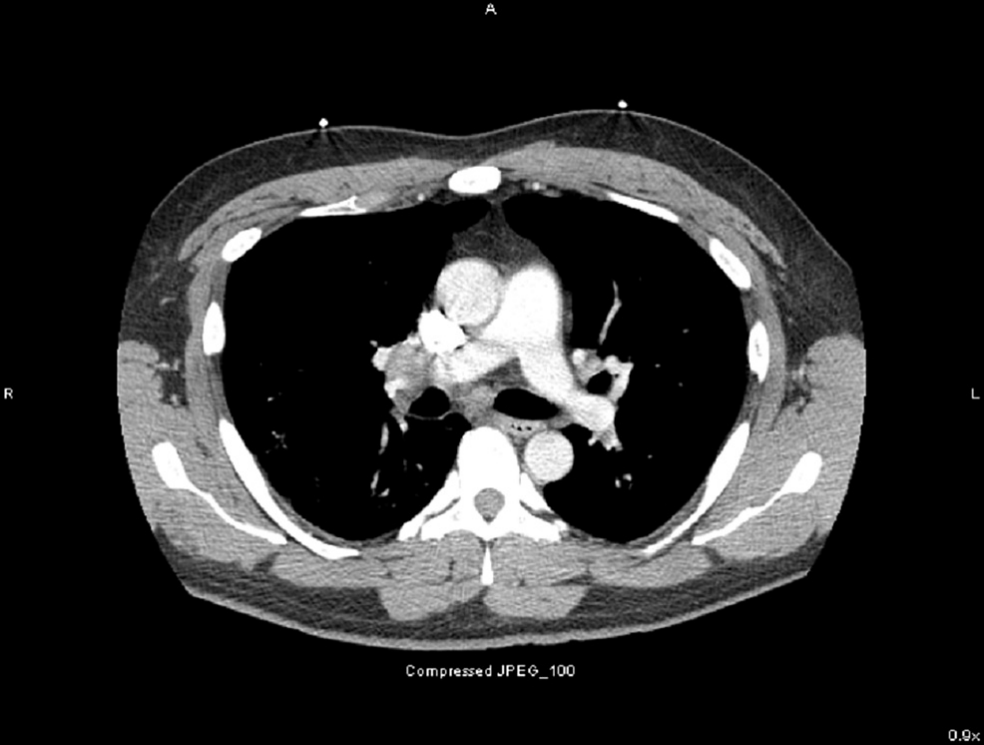
CT of the chest with subcarinal lymph node enlargement.

**Figure 3:**
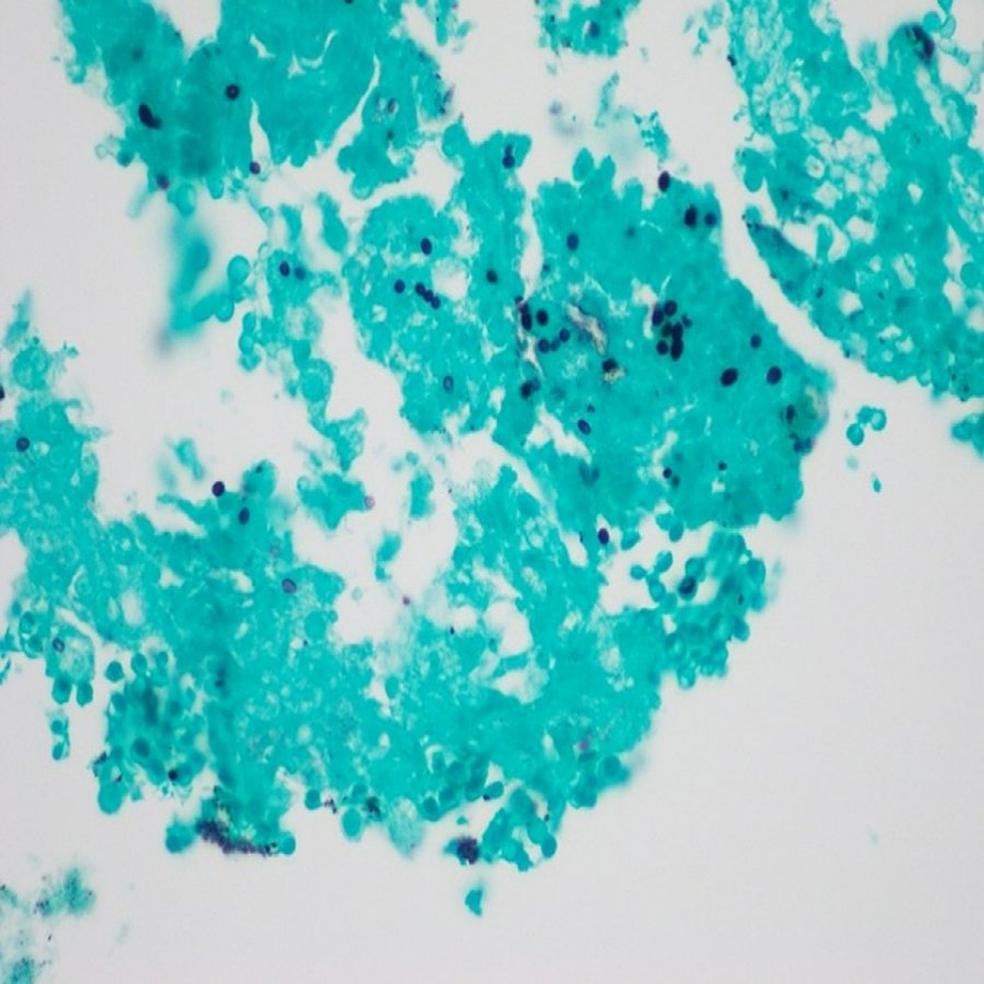
Grocott methenamine silver (GMS) stain with budding yeast.

A 50-year-old male that lived in the Ohio River Valley with a history of mild intermittent asthma well controlled on albuterol and fluticasone-salmeterol and no recent requirement of oral corticosteroids was admitted for daily cyclical fevers after COVID-19 pneumonia. One month before admission, the patient was treated on the medical-surgical floor for COVID-19 pneumonia with a five-day course of remdesivir and a 10-day course of dexamethasone. The patient recovered and he was discharged home without oxygen. Two weeks after discharge, he developed fever and myalgias. The patient had two polymerase chain reaction (PCR) COVID-19 testing that both were negative. On hospital admission, COVID-19 PCR testing was also negative. His blood work on admission revealed WBC 7.9 x 10^3^ uL, hemoglobin 11.8 g/dL, and platelets 146 x 10^3^ uL. A non-contrast chest computed tomography (CT) revealed interval improvement of ground-glass opacities, a new 3.9 cm mass-like density in the right upper lobe (RUL), mildly enlarged mediastinal lymphadenopathy, and moderate-severe hepatosplenomegaly (Figures 1, 2). The patient was started on vancomycin and cefepime and transitioned to linezolid, levofloxacin, voriconazole, and piperacillin-tazobactam after no improvement in his fevers as high as 39.9°C. The patient continued to be febrile daily until hospital day 14 despite antipyretics. He was first noted to have pancytopenia on hospital day 7 with a possible cause due to Linezolid which was stopped on hospital day six. His repeat blood work revealed WBC 2.8 x 10^3^ uL, hemoglobin 8.8 g/dL, platelets 50 x 10^3^ uL, and ferritin 2,934 ng/mL. The patient underwent bronchoscopy with endobronchial ultrasound and transbronchial needle aspiration of subcarinal lymph node and biopsy of the RUL mass. Fine needle aspiration of the subcarinal lymph node showed budding yeast suggestive of histoplasmosis on the Grocott methenamine silver (GMS) stain (Figure 3). The Histoplasma galactomannan urine antigen was positive at 1.6 ng/mL (reference range < 0.2 ng/mL). The patient’s serum cryptococcal antigen and antibody and blastomyces antibody were negative. The bronchial alveolar lavage and tissue biopsy of the right upper lobe mass isolated Histoplasma capsulatum. Bone marrow biopsy revealed extensive granulomatous inflammation consistent with disseminated histoplasmosis. His cyclic fevers ceased, and pancytopenia improved with amphotericin-B. He was discharged from the hospital one week after starting treatment with a plan of two weeks of therapy and transition to oral fluconazole.

## Discussion

Risk factors for most of the reported cases of disseminated histoplasmosis in immunocompetent patients include living in a rural setting due to the likely exposure to histoplasma in the soil [8]. Most of these patients experienced fever, weight loss, hepatosplenomegaly, lymphadenopathy, pancytopenia, elevated ESR, elevated hepatic transaminases, and had bone marrow involvement [8,9]. An immunocompromised state, including HIV/AIDS and chemotherapy-induced immunosuppression, is a known risk factor for disseminated histoplasmosis. There is a reported case of disseminated histoplasmosis in a patient with HIV/AIDS after COVID-19. This patient presented with an initial CD4 count of 113 cells/mm^3^ and a viral load of 38,503 RNA copies/ml. One week after the initial presentation, the patient was diagnosed with COVID-19 and with suspected disseminated histoplasmosis based on a positive histoplasmosis antigen and CT scan with lymphadenopathy and hepatosplenomegaly [6]. Another patient with underlying asthma and diabetes mellitus presented with COVID-19 pneumonia complicated by pulmonary embolism and prolonged hospitalization was diagnosed with pulmonary histoplasmosis based on positive Histoplasma capsulatum complement fixation titers [10]. 

Co-infecting pathogens with COVID-19 are well documented, and patients are more likely to die than those without a co-infection [2]. Initial reports included both viral and bacterial causes [2,11]. Fungal co-infections are increasingly reported after COVID-19 and are also known to increase mortality as well [12]. Most reported cases include aspergillus with a prevalence as high as 8.5% in retrospective studies and 9% in a meta-analysis [2]. Risk factors not mentioned previously for aspergillosis include antibiotics, particularly azithromycin (cumulative dose >/= 1500 mg) [13]. Many of these patients may be treated with Voriconazole to avoid the potentially nephrotoxic liposomal amphotericin B [12]. Candida, which is often isolated in the respiratory tract, is usually not thought to be pathogenic [1,2] and the reported cases of candida bacteremia were thought to be related to central line infections [14]. Other reported non-disseminated fungal co-infections include coccidioidomycosis, mucormycosis, and cryptococcosis [1,11].

Acute pulmonary histoplasmosis presents with either asymptomatic disease, flu-like symptoms, caseating or noncaseating granulomatous disease, or mediastinal adenopathy. Chronic pulmonary histoplasmosis presents with an either cavitary or noncavitary disease with associated nodules, infiltrates, and mediastinal lymphadenopathy. Disseminated disease is often seen in immunosuppressed patients. Manifestations can include skin involvement with polymorphic plaques, nodules, and erosions, gastrointestinal involvement with hepatosplenomegaly and colonic ulcerations, and bone marrow suppression [3,4].

Diagnosis of disseminated disease is complex as many tests take weeks. Cultures often take two to four but up to eight weeks with the greatest yield occurring in those with disseminated disease. Complement fixing antibodies may appear two to six weeks following acute infection and may persist for years following infection. Immunodiffusion antibody assay tests for the presence of M and H precipitin bands. M bands develop with acute infections and persist for months to years. H bands appear after an M band and may disappear earlier than M bands. These may indicate active histoplasmosis [3]. Antigen testing may be more effective in acute disease and immunocompromised patients who cannot amount an antibody response with improved sensitivity utilizing both serum and urine testing. A polymerase chain reaction is available but still needs further standardization [3,15].

Treatment for the mild asymptomatic acute pulmonary disease is often unnecessary. Moderate to severe acute pulmonary disease requires treatment with amphotericin B followed by azole antifungal therapy for weeks. Chronic pulmonary disease is treated with azole antifungal therapy for 12-24 months. The disseminated disease requires treatment with amphotericin B for one to two weeks followed by azole antifungal therapy for at least 12 months [7]. 

## Conclusions

We presented a case of disseminated histoplasmosis after COVID-19 pneumonia effectively treated with amphotericin-B and fluconazole. The patient was eventually discharged home with resolution of his cyclic fevers and pancytopenia. Fungal infections are increasingly reported in association with COVID-19 pneumonia. Although not able to prove, prolonged courses of dexamethasone may be a risk factor for disseminated fungal infections. Clinicians need to be aware of the possibility of disseminated fungal diseases even in immunocompetent patients.
